# Psychometric properties and factor structure of the 13-item satisfaction with daily occupations scale when used with people with mental health problems

**DOI:** 10.1186/s12955-014-0191-3

**Published:** 2014-12-24

**Authors:** Mona Eklund, Martin Bäckström, Aaron M Eakman

**Affiliations:** Department of Health Sciences, Occupational Therapy and Occupational Science, Lund University, PO Box 157, SE-221 00 Lund, Sweden; Department of Psychology, Lund University, Lund, Sweden; Department of Occupational Therapy Program, Colorado State University, Fort Collins, CO USA

**Keywords:** Factor analysis, CFA, Homogeneity, Psychiatry, Occupational therapy

## Abstract

**Background:**

In mental health care practice and research it is increasingly recognized that clients’ subjective perceptions of everyday occupations, such as satisfaction, are important in recovery from mental illness. Instruments thus need to be developed to assess satisfaction with everyday occupations. The aim of the present study was to assess psychometric properties of the 13-item Satisfaction with Daily Occupation (SDO-13) when used with people with mental health problems, including its internal consistency, factor structure, construct validity and whether the scale produced ceiling or floor effects. An additional question concerned if the factor structure varied whether the participants were, or were not, presently engaged in the activity they rated.

**Methods:**

The interview-based SDO-13 includes items pertaining to work/studies, leisure, home maintenance, and self-care occupations. Whether the person currently performs an occupation or not, he/she is asked to indicate his/her satisfaction with that occupation. The SDO-13 was completed with 184 persons with mental illness. Residual variables were created to remove the variation linked with currently performing the targeted occupation or not and to assess the factor structure of the SDO-13. The indicators of general satisfaction with daily occupations, self-esteem and global functioning were used to assess construct validity. The statistical methods included tests of homogeneity, confirmatory factor analysis and Pearson correlations.

**Results:**

The internal consistency was satisfactory at 0.79. A three-factor solution indicated that the construct behind the SDO-13 was composed of three facets; Taking care of oneself and the home, Work and studies, and Leisure and relaxation. The same factor structure was valid for both original scores and the residuals. An expected pattern of correlations with the indicators was mainly found, suggesting basic construct validity. No ceiling or floor effects were found.

**Conclusions:**

Taken together, the findings suggest the SDO-13 is a reliable and robust instrument that may be used to get an overview of the satisfaction people living with mental illness derive from their daily occupations.

## Background

In mental health care practice and research it is increasingly recognized that clients’ subjective perceptions of everyday occupations are important in recovery from mental illness [1,2], inform assessments of clients’ problems and assets, and serve a key role in evaluating clinical outcomes [3,4]. Occupation in this sense denotes the everyday activities that compose people’s lives and includes areas such as work, leisure, home maintenance, personal care and social interaction. Prior research has indicated that persons’ subjective perceptions of satisfaction with everyday occupations offer unique insights towards understanding personal well-being. For example, a study among people with mental illness showed that subjective perceptions of occupation were more closely associated to key outcomes such as quality of life and self-rated health, but also to psychosocial functioning as assessed by a professional, than were factors pertaining to actual doing, such as time use and activity level [5].

One of the subjective aspects of occupation in the latter study was satisfaction with daily occupations. Whereas occupational satisfaction is typically used to describe personal subjective evaluations of day to day engagement, and is often seen as an aspect of well-being [6], few have tried to define the concept. Yerxa [7] viewed satisfaction with occupations as an aspect of health, which she defined as “… an encompassing, positive, dynamic state of ‘well-beingness,’ reflecting adaptability, a good quality of life, and satisfaction in one's own activities” (p. 412). She further argued that satisfaction may be achieved through developing new capacities, adjusting the environment, fostering ambition, enhancing performance, etc., as long as such endeavors are compatible with one’s culture and personal beliefs. Citing Adolph Meyer, Yerxa stated that satisfaction is “doing and getting enough” (p. 413). This is in line with how satisfaction is viewed within other fields such as motivational and developmental psychology. McClelland [8] suggested, for example, that satisfaction is derived when a person’s life activities support his or her fundamental motivations. Furthermore, Self-Determination Theory developed by Ryan and Deci [9] postulates three innate needs (competence, autonomy and relatedness) that foster intrinsic motivation, i.e. when behavior occurs as a result of free choice and without any apparent external rewards, which in turn leads people towards seeking new challenges [9,10]. Satisfaction of needs related to intrinsic motivation is thus compatible with the occupational therapy and occupational science belief that humans have an innate drive to be active and seek competency and achievements and may be seen as another aspect of satisfaction with daily occupations.

Occupational satisfaction has also been linked with lifestyle balance [11–13], defined by Matuska and Christiansen [14] as “a satisfying pattern of daily occupation that is healthful, meaningful, and sustainable to an individual …” (p. 11). Morgan [15], when addressing occupational satisfaction, argued that occupations that are principally challenging and personally valued are those that generate satisfaction. Thus, according to these definitions, satisfaction with daily occupations is about doing and getting enough, doing valued things that align with one’s motivations and needs, and perceiving one has a balanced lifestyle.

A tool for screening people’s satisfaction with daily occupations was developed for use with people with mental illness some years ago [16]. That instrument, the Satisfaction with Daily Occupations (SDO) instrument, consisted of nine items addressing the areas of work, leisure, home maintenance and personal care. Those areas were targeted against the backdrop of theoretical occupational therapy literature, typically categorizing everyday occupations according to similar taxonomies [17–19]. The construct behind the SDO concerns people’s available occupations and the satisfaction they derive from those occupations, in accordance with how satisfaction with everyday occupation is defined above. The performance per se is not rated. Each item is responded to in two ways. First the respondents answer if they are presently engaged in that type of occupation or not (yes/ no). Regardless of whether they answer yes or no, they subsequently rate their satisfaction with the situation regarding the targeted occupation. No study has, however, investigated whether satisfaction with currently being engaged in an occupation is conceptually the same as satisfaction with not presently being engaged in that occupation.

The SDO was constructed because a gap among existing instruments was identified. The Canadian Occupational Performance Measure (COPM) [20] also assesses satisfaction with occupational performance, but a less time-consuming and more structured tool was needed for clinical and research purposes. The SDO was indeed found easy to understand and quick to administer by both occupational therapists and clients [3]. Another advantage with the SDO is that it has pre-defined items, reflecting common everyday activities, as opposed to the COPM where each respondent defines his or her own problematic activities. This makes the SDO suitable for aggregating data and making group comparisons. So far, the SDO has shown good content validity and test-retest reliability and the ability to discriminate between groups with differing severities of mental illness [3,21]. It has also been found useful with non-psychiatric groups, namely healthy women and women with scleroderma [22]. However, frequency distributions have indicated that most items yield responses in the upper part of the seven-point response scale, and additional items would be preferable to avoid possible ceiling effects [23]. In response to that, an extended test version with 13 items has been developed, with the intention of adding items that are less likely to give high ratings by people with mental illness. Those items were developed in dialogue with clinical occupational therapists that had extensive experience from using the SDO. The 13-item version was recently found reliable and valid in a Danish study [24].

The purpose of this study was to assess the psychometric properties of the SDO-13 when used with people with mental illness; this group was the initial target population when the SDO was originally developed. Part of the aim of this study was to establish whether the SDO-13 possessed acceptable internal consistency and construct validity and whether the scale produced ceiling or floor effects. The aim was also to investigate the factor structure of the SDO-13, including if the factor structure varied whether the participants were, or were not, presently engaged in the activity they rated.

## Methods

### Participants

Participants for this study were sought in the context of community-based psychiatry, among attendees at day centers for people with psychiatric illnesses. Attendees in nine districts, both urban and rural areas, were approached specifically for this study. Those who were in an acute phase of their illness, as assessed by the staff, were excluded, as were those who did not understand written and spoken Swedish. The others were invited to participate in the present study. After oral and written information from the researchers was provided, those who agreed to participate gave their written consent. In all, 184 attendees participated, corresponding to 50 per cent of those who were asked. In previous studies based on comparable samples, and when the data collection was not part of clinical routines, similar response rates have been obtained [25,26].

The day centers did not keep a register of diagnoses, but all participants were assessed as having a psychiatric disability, defined as a condition lasting more than two years and substantially hampering the person’s everyday life because of mental illness [27]. Nonetheless, participants’ offered self-reported diagnoses which were then grouped by a medical doctor who specialized in psychiatry into four larger diagnostic categories based on the ICD-10 system [28]. The diagnostic groupings were: 1 – schizophrenia and other psychotic disorders (interval F20 and affective psychoses from F30), 2 – mood and anxiety disorders (the remaining diagnoses from F30 plus interval F40), 3 – neuropsychiatric disorders (intervals F80 and F90) and 4 – other diagnoses (intervals F00, F10, F50-60). Socio-demographic characteristics and the diagnostic groupings are shown in Table 1. No analysis of representativeness was feasible for the present study, due to the registry routines in community-based psychiatry in Sweden.

**Table 1:** **Socio-demographic characteristics of the respondents (** ***n*** **= 184)**

**Characteristics**	**Details**
Age in years, mean (*SD*)	48.0 (10.6)
Gender, %	
Male	44
Female	56
Cohabitating, %	
Yes	17
No	83
Having children, %	
Yes	47
No	52
Educational level, %	
Not completed compulsory school	5
Completed compulsory school	30
Completed 6th form college	50
Completed undergraduate studies	15
Diagnostic group, %	
Psychoses (F20)	26
Mood and anxiety disorders (F30 + F40)	44
Autism/neuropsychiatric disorders (F80 + F90)	16
Other disorders (mostly F60 or not known)	13
Psychotropic medication, %	
Regularly/ when needed	80
Not at all	20

Note. *SD* = standard deviation.

### Instruments

#### The SDO-13

The original SDO was developed to briefly assess occupational areas of importance to clients in clinical practice, but also to be used as an outcome measure in clinical practice and research. The original nine-item SDO had four items related to work or studies, two pertaining to leisure-time occupations, two to home maintenance and repairs and one to personal care. The content of the nine-item SDO was discussed between a researcher and a panel of clinical occupational therapists. Three panel meetings with ten occupational therapists were held. Some of them participated at more than one meeting. The discussions indicated that additional questions were needed which addressed more advanced aspects of everyday activities, such as planning and organization of the domestic tasks and duties, taking care of others and, engaging in cultural leisure occupations and keeping physically and mentally fit. Another five items, one concerning leisure, two pertaining to home maintenance and two to personal care, were added to complete the 13-items test version. These items were developed to reflect occupations more challenging to perform and were added to reduce the likelihood of a ceiling effect in the SDO [23]. One original item was excluded, concerning attending a day center, because it specifies a specific type of support rather than an occupation. Table 2 lists each of the SDO-13 items; new items are indicated by italics and the deleted item is noted in bold. On the SDO used in this study, examples of the targeted activities were included to facilitate participants’ understanding. A seven-point scale was used for the satisfaction rating, ranging from extremely dissatisfied (=1) to extremely satisfied (=7). The panel of occupational therapists assessed this version as having an acceptable level of face validity as well as including more difficult items compared to the nine-item version of the SDO.

**Table 2:** **Item content of the 13-item test version of the satisfaction with daily Occupations Instrument (SDO-13)**

1	Presently employed or enrolled in college/folk high school
2	Working or enrolled in college/folk high school in past two months
3	Attending work training in past two months
4	Engaged in organized leisure occupations/hobbies at least once a week in past two months
5	Performing leisure occupations/hobbies on one’s own at least once a week in past two months
*6*	*Taking part in cultural occupations at least once a week in past two months*
7	Doing household work, such as cleaning and cooking, almost daily in past two months
8	Doing repairs and/or gardening in past two months
*9*	*Organizing and planning the household work in the past two months*
*10*	*Taking care of children, parent or other close persons at least once a week in the past two months*
11	Managing own personal hygiene on a daily basis
*12*	*Doing physical exercises at least once a week in the past two months*
*13*	*Doing activities to relax at least once a week in the past two months*
	**Attending a day center for meaningful activity in past two months**

Note. Italics indicate new items and bold text a deleted item.

### Measures for assessing construct validity

We used three measures to assess construct validity. These measures were chosen because they had varying degrees of similarity with the target construct as assessed by the SDO-13. One measure, a one-item estimate used previously to address general satisfaction with daily occupations [24], was expected to exhibit a strong relationship with the SDO-13. The second, addressing self-esteem [29], was anticipated to show a moderate relationship, and the third, concerning global functioning [30], was expected to show a weak correlation. Construct validity is often estimated in terms of correlations with other measures, and various terms are used depending on 1) whether an established and valid measure of the targeted construct exists; and 2) how theoretically similar the reference constructs are to the target construct. Since no established measure of satisfaction with daily occupations could be found, and it was important to assess associations with both similar and diverging constructs to obtain a relevant estimation of construct validity, convergent and discriminant validity were considered appropriate for the current study. Convergent validity is the agreement between constructs that according to theory should be more-highly associated with each other, whereas discriminant validity is the degree of agreement between constructs that should be less-highly associated or dissimilar according to theory. Another way of approaching discriminant validity is to employ different perspectives in ratings, such as comparing a score produced by a neutral rater with that given by the person him/herself; i.e., observer rating compared to self-report rating [31]. These differing perspectives on the same construct would produce at least partially divergent ratings, such as when both the observer and the respondent rate the respondent’s level of functioning. Alternatively, when self-report is used to assess partly differing constructs, for example the respondent’s ratings of his/her quality of life and level of functioning, similar ratings might be expected. In this latter case, a general subjective factor has been proposed to lie behind relationships between many types of self-reports [32]. It should therefore be expected that self-reports, although addressing differing constructs, will tend to be related. Based on reasoning related to 1) construct similarities, and 2) ratings based upon observer versus self-report, it was hypothesized that similarity with the construct behind the SDO-13 in both respects would lead to a strong correlation, similarity in one respect would lead to a moderate correlation, and no similarity in these two respects would lead to a weak correlation.

We used two instruments to assess convergent validity. The first was a one-item estimate of *general satisfaction with daily occupations* [24], developed specifically for the present study. The item was formulated as, “In general, how satisfied are you with your day to day activities?” and a five-point response format was used, from 1 = very dissatisfied to 5 = very satisfied. It was hypothesized that the SDO-13 would show a strong relationship with general satisfaction, since they target the same construct (i.e., satisfaction with daily occupations) and employ the same perspective on reporting (i.e., self-report). The limits set by Cohen [33] were used to estimate the strength of relationships. He proposed that correlations of 0.1 – 0.3 are weak, 0.3 – 0.5 are moderate and that relationships of 0.5 or more are strong.

The second instrument, *Rosenberg’s self-esteem scale* Rosenberg (RES) [29], was expected to exhibit a moderate relationship. Self-esteem measured by the RES has been found to reflect a relatively stable trait rather than objective functional status [34]. Further, although the SDO-13 and the RES address different constructs (the self and everyday activities), both measures have a subjective factor to them (esteem and satisfaction) based upon self-report. These were the reasons for hypothesizing a moderate relationship. The RES includes ten items with yes/no response alternatives. The final score indicates a balance between positive and negative self-esteem, varying from −1 (negative) to 1 (positive). The instrument has shown satisfactory psychometric properties [35].

The measure used to assess discriminant validity was the *Global Assessment of Functioning scale* (GAF). A professional (in this case a research assistant) rates a person’s global functioning on a scale from 0–100. GAF addresses the severity of the person’s mental illness in terms of two ratings; psychiatric symptoms and level of functioning (social, psychological and vocational) [36]. The lowest of these ratings constitutes the final GAF score. The GAF has been adopted as valuable for a wide range of systems and institutions [37]. Psychometric research on GAF has demonstrated that the instrument gives a valid assessment of psychiatric symptoms and social functioning and that it is reliable after brief rater training [38]. The research assistants were trained on how to perform the GAF ratings and followed an interview guide with questions regarding socio-demographic conditions, psychiatric symptoms, perceived severity of symptoms, sleep, suicidal ideation and employment situation. Good inter-rater reliability was indicated by an intra-class correlation of 0.86 when research assistants assessed three realistic video cases. The hypothesis for this study was that there would be a weak correlation between the GAF and the SDO-13. Research has found satisfaction with daily occupations to be unrelated with psychiatric symptoms but somewhat related with level of functioning in multivariate analyses [5]. Moreover, the GAF employs an observer rating (as compared to the use of self-report in the SDO-13).

### Procedure

The data collection was performed by research assistants who were not involved in the treatment of the clients and who had previous experience from data collection for research projects. Questions about socio-demographic factors were asked as well. The meeting took place in a secluded and quiet room at a participant’s day center.

### Data analyses

Internal consistency was tested by Cronbach’s alpha analysis, including corrected item-total correlations (CITC). The alpha value should be >0.70 to be considered as satisfactory when the instrument is used to compare groups [39], and the CITCs should be >0.20 to indicate a homogeneous scale [31]. Exploratory Factor Analysis, using Maximum likelihood (ML) to extract factors and employing Promax rotation, was used to investigate the factorial structure. The final model was tested by Confirmatory Factor Analysis defining the ratings as both continuous (estimation with ML) and ordinal variables (using weighted least squares means and variance adjusted [WLSMV] estimation). Since the participants rated their satisfaction on items whether they currently performed the activity depicted in the item or not, configural invariance was tested between a model estimation based on raw ratings and a model estimation based on ratings controlled for whether the activity was presently performed of not. Thus, the variation related with responding yes or no was removed from the ratings in the comparison data set. The remaining residuals composed a second data set, which was compared with the raw data set. This test revealed whether the factor structure varied when taking into account that some participants had not recently been engaged in the activities they rated.

Convergent and discriminant validity were estimated by Pearson correlations. Floor and ceiling effects were explored by means of frequency tables. A proportion of 5% or less of a sample with a maximum score (ceiling effect) or minimum score (floor effect) reflects effective measurement, whereas maximum or minimum scores exceeding 20% of a sample are considered to be substantive and indicate either a ceiling effect or floor effect [23,40]. The software used was the IBM SPSS Statistics 20.0 and Mplus version 7.11 [41].

## Results

### Internal consistency

A Cronbach alpha coefficient of 0.79 was obtained, which is within the limit for satisfactory internal consistency. All CITCs varied between 0.34 and 0.56, thus well above the lower limit of 0.20.

### Factor model

The SDO-13 item ratings were subjected to an exploratory factor analysis (EFA) with Promax rotation. The first three eigenvalues were 2.499 (19.2%), 2.561 (19.7%), and 0.744 (5.7%). Testing a model with only two factors gave a *χ*^2^ (df = 53) of 124.0 (p < 0.001) and testing a model with three factors resulted in a *χ*^2^ (42) of 58.73, p > .01. After rotation, the eigenvalues were 2.264, 2.39, and 2.634. Factor 3 correlated moderately high with factor 1, r = .30, and with factor 2, r = 0.49, while the correlation between factor 1 and factor 2 was low, r = 0.06.

Confirmatory factor analysis (CFA) using the MPLUS program was used to evaluate the fit of the EFA models. When the indicators were defined to be continuous, the fit of the three-factor model was acceptable, *χ*^2^(62) = 109.1, RMSEA = 0.06, CFI = 0.93. The three-factor model was compared with the one-factor and two-factor models. It was clearly better than a one-factor model, *χ*^2^(65) = 447.2, RMSEA = 0.18, CFI = 0.44, and a two-factor model *χ*^2^(65) = 713.5, RMSEA = 0.09, CFI = .86. To test if the factor structure was dependent on the items' distributions, the three models were also tested when all items were defined to be ordinal. The one-factor model had the worst fit *χ*^2^(65) = 160.4, RMSEA = 0.22, CFI = .71, followed by the two-factor: *χ*^2^(64) = 213.2, RMSEA = 0.13, CFI = 0.93, chi2(62) = 153.4. The three-factor: RMSEA = 0.09, CFI = 96, fit the data best. See Table 3. Taken together, these analyses clearly support the three-factor model and show that this model was not dependent on distributional issues.

**Table 3:** **Loadings and factor correlations from confirmatory factor analysis**

	**Factors continuous indicators**			**Factors ordinal indicators**		
	**F1**	**F2**	**F3**	**F1**	**F2**	**F3**
Factor loadings						
Item 7: household work	.747			.766		
Item 8: repairs/gardening	.455			.533		
Item 9: organizing household	.781			.809		
Item 11: personal hygiene	.531			.617		
Item 13: relaxation	.568			.641		
Item 1: employed/registered student		.840			.857	
Item 2: presently enrolled in work/studies		.953			.972	
Item 3: work training		.639			.737	
Item 4: organized leisure/hobbies			.387			.436
Item 5: autonomously performed leisure/hobbies			.769			.764
Item 6: cultural activities			.681			.730
Item 10: caring for others			.399			.480
Item 12: physical activity			.508			.553
Factor correlations						
F2	.122			.168		
F3	.608	.328		.699	.394	

To test whether the factor structure was invariant between ratings of items linked with a presently performed activity compared to when it was not presently performed, 13 new residual variables were extracted, taking out influence from activity being present/not present. Models based on the original SDO-13 items and the SDO-13 items’ residuals were tested to investigate if the three-factor model was configurally different in the two data sets. Configural invariance between the two data sets was tested by comparing a model with all indicator loadings defined to be equal with a model with freely estimated loadings. The factor loadings of both models were restricted to zero and the intercepts of the indicators were freely estimated. The models were not found to be significantly different, fit for the restricted model was *χ*^2^(134) = 243.2, RMSEA = 0.067, CFI = 0.92, and for the free model *χ*^2^(124) = 226.5, RMSEA = 0.067, CFI = 0.93, Δχ^2^(Δ10) = 16.7, p > 0.05. In addition, the Akaike information criterion for the free model (18230) was somewhat lower than for the restricted (18233). To test whether there was also configural invariance when data were based on ordinal variables, some of the indicators had to be collapsed to 6 instead of 7. The test did not reveal any significant differences between the data set based on original scores and that based on residuals.

To summarize, the SDO-13 was found to have three partly independent factors. The first had high loadings to variables measuring home maintenance and personal care, the second to variables measuring work-related occupations and the third to variables measuring leisure and relaxation. The models were also robust to scale problems, e.g. that the variables were ordinal and restricted in range (1–7) and that the distributions were non-normal. Moreover, the model was not dependent upon participants’ ratings related to whether or not they presently performed the activity or not.

### Construct validity

The correlation between the SDO-13 and general satisfaction with daily occupations was r = .44 (p < .001) and that to self-esteem was r = .36 (p < .001). Both of these were in the range of a moderate association. The correlation with global functioning was weak, r = .21 (p = .005).

### Floor and ceiling effects

Table 4 shows the proportions of participants using the lowest and the highest SDO-13 response alternatives, respectively, when making their ratings. Items 6, 10, 11 and 12 attracted the highest proportions of maximum ratings, around 40%. The minimum ratings were used less often, <15% for all items. With respect to the SDO-13 scale as a whole, with a possible range of 13 to 91, nobody scored lower than 25 and two individuals (1%) reached the maximum score. The participants’ mean rating (SD) was 65 (12.5).

**Table 4:** **Distribution in percentage on the lowest and highest rating alternative for the 13 items**

		**Lowest rating (1)**	**Highest rating (7)**
1	Presently employed or enrolled in college/folk high school	13	17
2	Working or enrolled in college/folk high school in past two months	14	20
3	Attending work training in past two months	10	23
4	Engaged in organized leisure occupations/hobbies at least once a week in past two months	11	22
5	Performing leisure occupations/hobbies on one’s own at least once a week in past two months	7	33
6	Taking part in cultural occupations at least once a week in past two months	2	41
7	Doing household work, such as cleaning and cooking, almost daily in past two months	5	20
8	Doing repairs and/or gardening in past two months	5	28
9	Organizing and planning the household work in the past two months	3	25
10	Taking care of children, parent or other close persons at least once a week in the past two months	2	38
11	Managing own personal hygiene on a daily basis	1	39
12	Doing physical exercises at least once a week in the past two months	8	44
13	Doing activities to relax at least once a week in the past two months	4	31

## Discussion

The SDO-13 showed satisfactory internal consistency reliability and all items showed satisfactory item-total correlations. Its factor structure was a critical issue, since responding yes and no, respectively, to currently being involved in an occupation might produce conceptually differing types of satisfaction. This was not the case, however. The factor structure obtained by EFA based on the raw satisfaction scores was confirmed in CFA when the variation linked with responding yes or no was removed. Three factors, seen as facets of the construct behind the SDO-13, were obtained; the first concerned Taking care of oneself and the home, the second Work and studies, and the third Leisure and relaxation. The first and third were highly correlated (r > .60), whereas Work and studies showed low correlations to both of these two. Whether it would be fruitful to develop subscales of the SDO-13 is an issue for future research, and would require replication of the factors identified here.

The hypotheses regarding relationships between the SDO-13 and the measures used to assess construct validity were mostly confirmed; they were in the expected order when observing the sizes of the correlation coefficients. The correlation between the SDO-13 total score and general satisfaction with daily occupations, however, only reached r = 0.44. Since both the targeted construct (occupational satisfaction) and the rater perspective (self-report) were the same, a strong association, >.50 according to Cohen [33], was expected. Convergent validity was thus not fully confirmed. The associations with self-esteem (r = 0.36; moderate) and global functioning (r = 0.21; weak) were as expected. As hypothesized, the results indicated that self-esteem displayed a lower correlation with the SDO-13 than did the single occupational satisfaction item. Both measures were self-report though differed in terms of their underlying construct. Further, the relationship between the SDO-13 and global functioning was weak and less than the relationship with self-esteem, which was another finding in line with the proposed hypotheses. In this latter relationship between the SDO-13 and global functioning the measures varied both in terms of the underlying construct and the approach to assessment (i.e., self- versus other-report). In sum, the pattern of associations in terms of convergent and discriminant validity reflect nearly full support of the hypotheses proposed for this study.

These findings concerning construct validity are in agreement with those obtained for the Danish version of the SDO-13 when used with two samples, a healthy group and a group of asylum seekers [24]. However, the construct validity findings were not quite congruent with those found for the original nine-item SDO when used with people with mental illness, where a closer relationship to global functioning was found [21]. The fact that the SDO-13 shows similar properties in different language versions and with various target groups, but deviates somewhat from findings regarding the nine-item SDO in the same language and with the same target group, indicates that the constructs behind the SDO-13 and the nine-item SDO are not identical.

No floor or ceiling effects were identified within the SDO-13 when using the proposed criteria [23,40]. Although some single SDO-13 items attracted a large proportion of maximum ratings, only 1% of the participants reached the maximum total score. The items introduced to represent more challenging occupations did not function as such, however, which is obvious when comparing Table 2 and Table 4. The new items are among those where most participants used the maximum score. The face validity, as assessed by the clinical occupational therapists, was thus not confirmed in this respect. Nevertheless, this did not lead to a ceiling effect in the scale as a whole and the new items broadened the range of daily occupations represented in the SDO-13. The broader range may have been important in the findings of similar properties in varying samples, such as the healthy Danes and the asylum seekers in a previous study [24] and the current sample of people with mental illness.

### Study limitations

A one-item indicator was used to test convergent validity, and a single item is always more likely than a multi-item scale to produce error variance. Although single-item indicators tend to offer acceptable levels of reliability and validity [42], a composed instrument, such as the COPM [20], which includes an occupational satisfaction scale, would have been preferable. On the other hand, the COPM addresses only activities that are seen as problematic by the respondent. This makes it an unsuitable candidate for comparison with the SDO-13, so the best option at hand was used. Still, convergent and concurrent validity of the SDO-13 needs to be further investigated. Other properties, such as re-test stability, need to be addressed as well. Furthermore, the present sample, consisting of people with mental illness, was fairly homogeneous. The Danish version of the SDO-13 was found to be psychometrically sound in a healthy sample and among asylum seekers [24], but also the Swedish version needs to be investigated for psychometric properties in other samples.

## Conclusion

It seems safe to conclude that the SDO-13 is a homogeneous scale which addresses satisfaction in the facets of Taking care of oneself and the home, Work and studies, and Leisure and relaxation. These three factors were identified as stable, regardless of whether or not the participant currently performed the occupation in question. Initial discriminant validity was established, but further evidence of construct validity is needed, particularly convergent validity. This may require using a more reliable measure of satisfaction with occupations than the one-item estimate used in the current study. No ceiling or floor effects were evident, although there was a clear tendency of participants to use the maximum ratings more often than minimum ratings. Taken together, the findings suggest the SDO-13 is a reliable and robust instrument that may be used to get an overview of the satisfaction people derive from their daily occupations.
